# Comparative Assessment of Diverse Green Manure Species for Enhancing Soil Quality, Microbial Communities, and Earthworm Growth in Fallow Paddy Fields

**DOI:** 10.3390/microorganisms14040870

**Published:** 2026-04-12

**Authors:** Lijuan Sun, Zhenni Zhao, Qin Qin, Yafei Sun, Shiyan Yang, Xiaofeng Jiang, Zhenglong Wang, Jun Wang, Yong Xue

**Affiliations:** 1 ECO-Environment Protection Research Institute, Shanghai Academy of Agricultural Sciences, Shanghai 201403, China; sunliuliu2012@126.com (L.S.); zhyll6@163.com (Z.Z.); qinqin19870987@126.com (Q.Q.); sunsunsun_cool@126.com (Y.S.); 20220703@saas.sh.cn (S.Y.); jiangxf@saas.sh.cn (X.J.); 2 Shanghai Low Carbon Agricultural Engineering Technology Research Center, Shanghai 201403, China; 3 School of Chemical and Environmental Engineering, Shanghai Institute of Technology, Shanghai 201418, China; 4 Shanghai Right Way Environmental Protection Technology Co., Ltd., Shanghai 201208, China; qazwsx20411@163.com

**Keywords:** earthworm, enzyme activities, green manure, paddy fields, paddy soils, ryegrass, soil quality

## Abstract

The excessive use of chemical fertilizers is a primary driver of soil degradation in agricultural systems. Planting green manure during fallow periods offers a sustainable alternative for soil conservation. The present study investigated the effects of different green manure cropping systems (Ryegrass (TR), Chinese milk vetch (TM), and Spinach (TS)) on soil physicochemical properties, biological activity, and microbial communities, compared to a control (CT). Results demonstrated that green manure treatments significantly enhanced soil fertility by increasing the content of soil organic matter (SOM), available nitrogen (AN), available phosphorus (AP), and available potassium (AK). Notably, the TR treatment increased SOM, AN, and AP by 23.0%, 60.0%, and 44.6% (*p* < 0.05), respectively. Concurrently, key soil enzyme activities (urease, dehydrogenase, catalase) were significantly boosted (*p* < 0.05), with TR showing the most pronounced effect. Earthworm indicators (such as earthworm biomass and abundance) were significantly higher in the Ryegrass plots (*p* < 0.05). Microbial analysis revealed that TM enhanced bacterial diversity, whereas TR increased fungal richness (*p* < 0.05). Beneficial bacterial phyla, particularly Proteobacteria, exhibited a marked increase under the TM and TR treatments, while the fungal community underwent a favorable shift. Consequently, a significant elevation was observed in the overall Soil Quality Index (SQI) across all green manure treatments. Notably, the TR treatment resulted in a substantial 150% increase. In summary, ryegrass emerged as the most effective treatment in enhancing soil fertility, biological activity, and microbial diversity, underscoring its considerable potential as a green manure for sustainable soil management during fallow periods in paddy fields.

## 1. Introduction

Globally, the escalating demand for food production has led to intensive agricultural practices, often characterized by the excessive application of chemical fertilizers. The reliance on synthetic agricultural inputs has inadvertently contributed to extensive soil degradation, encompassing soil compaction, nutrient imbalances, diminished biodiversity, and increased agricultural non-point source pollution. This poses a substantial threat to long-term agricultural sustainability and ecological health [1]. In many rice-producing regions, particularly in Asia, the conversion of paddy soils to a fallow state entails both challenges and opportunities for soil restoration and enhancement [2]. Addressing these issues requires innovative and eco-friendly farming practices. Green manure is an important source of organic fertilizer for paddy fields [3]. Traditional agricultural production in China has predominantly emphasized the utilization of organic fertilizers to enhance soil fertility and augment crop yields. The application of green manure can enhance soil nutrients, especially organic matter, available potassium, available phosphorus, and alkali-hydrolyzed nitrogen, thereby improving crop yield and quality [4,5,6]. Planting green manure during the fallow period of paddy fields is an important measure for soil conservation [7].

Earthworms, functioning as ecosystem engineers, predominantly consume organic matter and microbial communities present on the soil surface and throughout the soil profile. The influence of earthworms on soil structure and fertility has been extensively documented in the scientific literature [8,9]. Earthworms play a crucial role in accelerating straw decomposition by enhancing microbial activity, fragmenting organic matter, and promoting nutrient cycling [10]. Their burrowing behaviors enhance soil aeration and water retention, thereby promoting the in situ decomposition of crop residues and supporting agricultural waste recycling and soil conservation efforts [11,12].

The development and hatching of earthworms are influenced by conditions such as relative humidity, pH, temperature, feed types, and relative density [13]. Therefore, planting green manure during the fallow period of paddy fields can provide a favorable habitat for earthworm growth, which is conducive to their colonization and enhances soil fertility. Furthermore, the incorporation of green manure supplies earthworms with nutrien-rich organic matter and favorable microhabitats, thereby enhancing their reproductive success and population density. The expanded earthworm population accelerates straw decomposition through the synergistic effects of multiple biological mechanisms (e.g., burrowing, ingestion, and digestion), while concurrently enhancing microbial activity and soil organic matter turnover. These processes enhance soil structure and promote the long-term sustainability and fertility of agricultural soils [14].

At present, there is a limited amount of research and application on the creation of habitats for earthworm farming in paddy fields. In the establishment of habitats for earthworm farming in paddy fields, the habitat constitutes the ecological backbone of the paddy field agricultural system and serves as the foundational network for earthworm cultivation. Investigating techniques for establishing optimal habitats for earthworm cultivation in paddy fields holds substantial practical significance. It ensures the self-reproductive capacity and viability of earthworms within the paddy system, enhances straw-processing productivity, and fulfills the ecological service provision of the production site.

Given the pivotal role of green manure in augmenting soil fertility, alongside the contribution of earthworms to organic matter decomposition and nutrient cycling, the primary objective of this research is to conduct a systematic assessment of the effectiveness of distinct green manure cropping systems in enhancing soil health and sustainability throughout the paddy fallow period. Therefore, we hypothesize that the cultivation of specific green manure crops, particularly Ryegrass, during the fallow period of rice paddies may substantially enhance soil fertility, biological activity, and microbial diversity. These improvements are expected to significantly elevate overall soil quality and promote earthworm growth, thereby contributing to sustainable agricultural practices. To test this hypothesis, the present study specifically aims to: (1) examine the impacts of distinct green manure cropping systems implemented during the paddy fallow period on soil physicochemical properties, with particular emphasis on nutrient availability—specifically (organic matter, nitrogen, phosphorus, and potassium—and to elucidate the mechanisms by which selected plant species influence soil quality; (2) evaluate the responses of earthworm abundance and biomass to green manure amendment, and assess their relationships with soil quality indicators; and (3) characterize shifts in soil bacterial and fungal community composition and diversity, and identify the microbial drivers associated with green manure-induced alterations in soil quality. By elucidating the synergistic effects of green manure and earthworms on soil quality, this study aims to establish practical guidelines for eco-friendly agricultural practices that enhance soil biodiversity, promote carbon sequestration, and ensure long-term agricultural sustainability.

## 2. Methods and Materials

### 2.1. Site Description

The experiments were performed at the Samsung Experimental (12°33′47″, 31°41′20″) on Chongming Island, Shanghai, China. As described in our previous research, the area is situated in the northern subtropical region and features a typical subtropical monsoon climate, with an annual mean temperature of 15.3 °C [15]. The experimental soil is a silty clay loam, classified as a leached paddy soil; the soil parent material is coastal sediments (the soil morphology, soil section photo, and soil horizon sequence can be referred to in the Supporting Information).

In the experimental field, seven plots were laid out, each measuring 6 m × 40 m. The geomembrane was carefully installed vertically along the boundaries of each plot, extending from the soil surface down to a depth of approximately 50 cm, to prevent exchange of water and nutrients and to stop earthworms from escaping. After paddy rice was harvested, the rice straw was chopped into pieces about 6 cm long and thoroughly incorporated into the soil by deep plowing with agricultural machinery. Then, each field plot was inoculated with juvenile earthworms (*Pheretima guillelmi*) at a density of 750 kg per hectare.

Ryegrass (*Lolium perenne* L.), Chinese milk vetch (*Astragalus sinicus* L.), and spinach (*Spinacia oleracea* L.) were selected as green manure crops. The seeding rate for each crop species was determined in accordance with local agronomic conventions. A control treatment was incorporated in which no green manure seeds were sown. The abbreviations for the above four treatment groups were TR, TM, TS, and CT, respectively. All the seeds were bought from Zhejiang Wuwangnong Seed Industry Co., Ltd. (Hangzhou, China). After straw chopping and returning to the field, green manure seeds were manually and evenly broadcast into the paddy fields at a specific sowing density. Conventional water and fertilizer management was maintained. During the green manure growth period, soil moisture was maintained at approximately 70% of field capacity through regular irrigation to ensure optimal plant growth and microbial activity. After the green manure had grown for 4 months, soil samples were collected, and the density and weight of earthworms were investigated and analyzed.

Earthworm Survey Protocol: A 60 cm × 60 cm iron frame was randomly placed in the field. Using a shovel, the top 0–20 cm layer of soil within the frame area was gently excavated. The soil on the shovel was then carefully dislodged, and all earthworms within the quadrat were collected. The number of earthworms was counted and recorded, and their weight was measured on-site. Within each plot, three survey quadrats were established as replicates.

### 2.2. Soil Sample Analysis

Soil samples for the experiment were collected from the topsoil layer (0–20 cm depth), which represented the plough layer and was most actively influenced by agricultural practices. The soil sample was divided into three portions. One portion was used for analyzing physicochemical properties including pH, soil organic matter, alkali-hydrolyzable nitrogen, available potassium, and available phosphorus. Another portion was used for assaying soil urease, cellulase, catalase, and dehydrogenase activities. The remaining portion was used for characterizing the soil microbial community structure and diversity.

### 2.3. Measurements and Methods

Soil pH was measured in a soil:water suspension (1:2.5) after 30 min of shaking at 25 °C using a pH meter (METTLER TOLEDO). Soil organic matter was measured using the potassium dichromate oxidation method [16]. Available N was determined using the alkali diffusion method and incubation at 40 °C for 24 h [17]. Available phosphorus was extracted with 0.5 mol/L sodium bicarbonate and determined by molybdenum antimony anticolorimetry, while available potassium was extracted with 1 mol/L ammonium acetate and determined by a flame photometer [18]. The soil enzyme activity was determined using an assay kit (Nanjing Jice Biotechnology Co., Ltd., Nanjing, China). Fresh soil samples were air-dried naturally and passed through a 30–50 mm mesh sieve. Urease, cellulase, catalase, and dehydrogenase activities were determined following the manufacturer’s instructions. Urease activity was determined by quantifying the ammonia released from urea hydrolysis. The ammonia reacts with phenol and sodium hypochlorite to form a blue indophenol complex, which is then measured colorimetrically at 630 nm. Cellulase activity was assayed by measuring the amount of reducing sugars released from carboxymethyl cellulase (CMC) hydrolysis using the dinitrosalicylic acid (DNS) method, with absorbance measured at 540 nm. Catalase activity was determined by measuring the consumption of hydrogen peroxide. The residual hydrogen peroxide reacts with ammonium molybdate to form a yellow complex, which is subsequently quantified spectrophotometrically at 405 nm. Dehydrogenase activity was determined by measuring the reduction of 2,3,5-triphenyltetrazolium chloride (TTC) to triphenylformazan (TPF), which was extracted with methanol and measured spectrophotometrically at 485 nm.

### 2.4. Determination of Soil Microorganisms

DNA was extracted from fresh rhizosphere soil samples using the E.Z.N.A.™ Mag-Bind Soil DNA Kit (OMEGA, USA; Shengong Biotechnology, Shanghai, China) following the manufacturer’s protocol [19]. The V3–-V4 hypervariable region of the bacterial 16S rRNA gene was amplified employing universal primers 341F (5′-CCTACGGGNGGCWGCAG-3′) and 805R (5′-GACTACHVGGGTATCTAATCC-3′). The ITS1F–ITS2 region of the fungal ITS was amplified employing primers ITS1F (CTTGGTCATTTAGAGGAAGTAA) and ITS2 (GCTGCGTTCTTCATCGATGC). PCR amplification was performed according to Tang et al. with minor modifications. PCR reactions (30 μL) contained 10–20 ng of template DNA. Thermal cycling conditions comprised initial denaturation at 94 °C for 3 min, followed by 5 cycles of denaturation at 94 °C for 30 s, annealing at 45 °C for 20 s, and extension at 65 °C for 30 s; subsequently 20 cycles of denaturation at 94 °C for 30 s, annealing at 55 °C for 20 s, and extension at 72 °C for 30 s; with a final extension at 72 °C for 5 min.

Amplicon DNA concentration was quantified utilizing the Qubit 3.0 DNA Assay Kit (Thermo Fisher Scientific, Waltham, MA, USA; Shengong Biotechnology, Shanghai, China). Raw sequence reads containing adapter and barcode sequences were processed to discard low-quality reads. Overlapping paired-end reads were assembled using PEAR software (version 0.9.11). The merged sequences underwent quality filtering via the QIIME pipeline, eliminating sequences containing ambiguous bases (‘N’) or low-complexity regions. Chimeric sequences were subsequently identified and removed. The resultant high-quality sequences were clustered into operational taxonomic units (OTUs) at a 97% similarity threshold employing UPARSE. Representative sequences from each OTU were taxonomically classified through alignment against the SILVA reference database.

### 2.5. Calculation of the Soil Quality Index (SQI)

Consistent with established methodologies in the literature, principal component analysis (PCA) was employed to analyze the soil physicochemical properties dataset [20]. Initially, a comprehensive dataset encompassing soil physicochemical parameters including soil organic matter (SOM), alkaline-hydrolyzable nitrogen (AN), available phosphorus (AP), readily available potassium (AK), and soil pH was established through standardized analytical protocols. To eliminate dimensional heterogeneity and ensure metric comparability, extreme-value normalization was implemented to derive dimensionless standardized scores (*Si*). The pH parameter, conforming to a “lower-is-optimal” paradigm, was transformed via Equation (1), whereas the remaining positively oriented indicators, adhering to a “higher-is-optimal” rationale, were standardized through Equation (2) [21].

(1) $$\mathrm{Si}\prime =\frac{X_{max}-\mathrm{Xi}}{X_{max}-X_{\min}}$$

(2) $$\mathrm{Si}\prime \prime =\frac{\mathrm{Xi}-X_{\min}}{X_{max}-X_{\min}}$$

In the equations, *Si* (*Si′* and *Si″*) denotes the standardized score of the ith indicator following transformation, whereas *X_i_*, *X_max_*, and *X_min_* represent the measured value, maximum value, and minimum value of the ith indicator, respectively.

Subsequently, principal components (PCs) were extracted based on the criterion of eigenvalues exceeding unity. The cumulative variance contribution rate was employed as the criterion for information retention, and the communality (*hi*^2^) of each indicator on the principal components was calculated according to Equation (3) [22].

(3) $${\mathrm{hi}}^{2}=\sum_{j=1}^{m}{L_{\mathrm{ij}}}^{2}$$

In the equation, *L_ij_*^2^ represents the squared factor loading of the ith indicator on the jth principal component, and m denotes the number of retained principal components.

According to the principal component analysis, the communalities of each indicator were normalized using Equation (4), thereby deriving the weight (*Wi*) of each indicator in the Soil Quality Index (SQI) [23].

(4) $$\mathrm{Wi}=\frac{{\mathrm{hi}}^{2}}{\sum_{k=1}^{n}{h_{k}}^{2}}$$

In the equation, *Wi* represents the weight assigned to the ith indicator, and n denotes the total number of indicators.

Finally, the weights (*Wi*) were multiplied by the standardized indicator scores (*Si*), which ranged from 0 to 1, and the Soil Quality Index (SQI) was subsequently calculated according to Equation (5) [24].

(5) $$\mathrm{SQI}=\sum_{i=1}^{n}\mathrm{Wi}\times \mathrm{Si}\;$$

### 2.6. Statistical Analysis

Statistical analyses were conducted using SPSS 25.0 (IBM Corp., Armonk, NY, USA). One-way analysis of variance (ANOVA), combined with Duncan’s multiple range test, was employed to evaluate differences in soil quality, enzyme activities, fungal richness, and diversity among experimental treatments at a significance level of *p* < 0.05. The data in the figures represent the mean ± standard deviation of three replicates. Principal component analysis (PCA) was performed to assess treatment effects on soil properties. Partial least squares discriminant analysis (PLS-DA) was utilized to analyze metabolic discrepancies across soil samples from the three distinct treatments. Mantel tests were applied to examine the influences of soil enzyme activities and fungal community composition on soil quality and earthworm biomass. Random forest models, implemented via the “vegan” package in R software (version 4.3.3) and accessed through the RStudio interface (version 1.3.1073), were employed to predict key soil variables governing soil quality and earthworm biomass. Linear regression analysis was used to assess the relationship between earthworm biomass and soil quality.

## 3. Results

As presented in Table 1, the cultivation of green manure crops exerted a relatively minor influence on soil pH. The values of the three green manure species treatments ranged from 8.10 to 8.31, demonstrating a tendency to be higher than that of the control (CT, 7.88 ± 0.25). The TR treatment (Ryegrass) induced the most significant alteration, registering the highest pH value and a 5.5% increase compared to the CT treatment. Regarding soil nutrients, green manure cultivation notably enhanced several indicators. In comparison with CT, the TR treatment was the most efficacious, increasing soil organic matter (SOM) by 23.0%, available nitrogen (AN) by 60.0%, and available phosphorus (AP) by 44.6%. Furthermore, all green manure treatments notably elevated the concentration of available phosphorus (AP) in the soil. Regarding available potassium (AK), an upward trend was detected in the TS and TM treatments. Overall, the TR treatment proved to be the most efficacious green manure for enhancing available nitrogen (AN) and soil organic matter (SOM) in the soil, with both of these critical parameters exhibiting a significant increase relative to the CT group.

**Figure 1:**
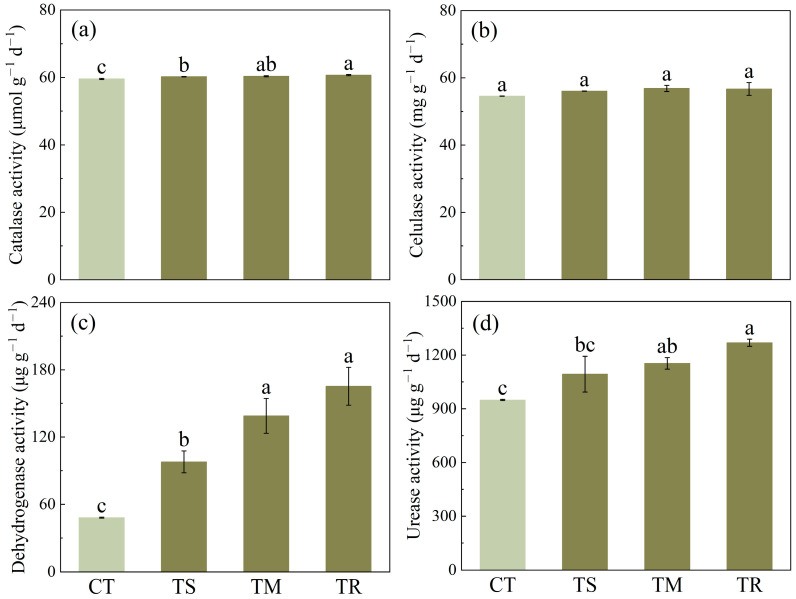
Activities of soil catalase (**a**), cellulase (**b**), dehydrogenase (**c**) and urease (**d**) under different treatments. Properties of soil under different treatments (CT, control with no green manure; TS, Spinach treatment; TM, Chinese milk vetch treatment; TR, Ryegrass treatment). Values are means ± standard deviation. Values followed by different letters within a column indicate significant difference (*p* < 0.05) in Duncan’s test.

As illustrated in Figure 1, the comprehensive assessment of four soil enzyme activities revealed that different green manure species resulted in significant variations in soil biochemical functions. The soil catalase activity was significantly elevated across all green manure treatments compared to the control (CT) group. Among these treatments, the TR treatment exhibited the greatest activity, which was significantly higher than that observed in the TS treatment. Soil cellulase activity was not significantly influenced by any green manure treatment. All treatments led to a significant increase in soil dehydrogenase activity (*p* < 0.05). Among the treatments, TM and TR exhibited the most pronounced effects, with activity levels increasing by 263% and 270%, respectively, relative to the CT group. The TS treatment also resulted in a substantial increase, exceeding 110%. Each treatment exerted significant effects on soil urease activity. The TR treatment resulted in a markedly significant increase in soil urease activity, whereas no significant differences were detected between the TM and TS treatments. These findings collectively indicate that, within the context of this soil enzymatic activity investigation, ryegrass represents the most efficacious green manure species for enhancing soil nitrogen cycling and overall microbial activity.

**Figure 2:**
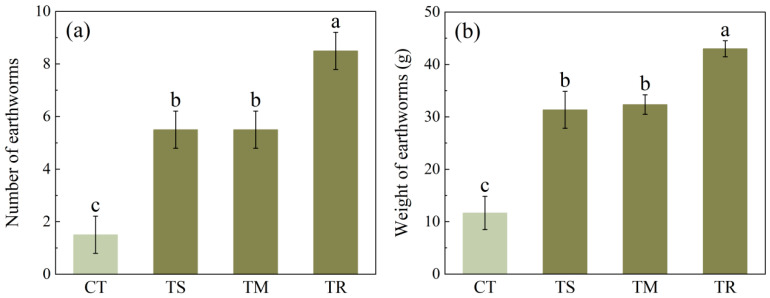
(**a**) The number of earthworms in each sampling quadrat underin different treatments. (**b**) The weight of earthworms in each sampling quadrat under different treatments. Properties of soil under different treatments (CT, control with no green manure; TS, Spinach treatment; TM, Chinese milk vetch treatment; TR, Ryegrass treatment). Values are means ± standard deviation. Values followed by different letters within a column indicate significant difference (*p* < 0.05) in Duncan’s test.

Figure 2 illustrates the influence of diverse treatments on both the quantity and biomass of earthworms within the sampling quadrats. Following the application of green manure, both earthworm abundance and biomass demonstrated significant increases, whereas the control group exhibited the lowest values for both parameters. Specifically, earthworm abundance in the TR treatment was significantly greater than that in the control and in the other treatments. A similar trend was observed in earthworm weight. The TR group exhibited a significantly greater earthworm weight compared to the control and other treatment groups, indicating enhanced accumulation of earthworm biomass under these conditions. In the TS and TM treatment groups, both earthworm abundance and biomass in the soil were significantly higher than those in the CT group; however, no statistically significant difference was detected between these two treatments, and their efficacy was lower than that of the TR treatment. Collectively, these data suggest that green manure treatments effectively promote earthworm populations and biomass in soil.

Table 2 shows the alpha diversity indices of bacterial and fungal communities in soil under different treatments. The bacterial Chao1 values (an estimator of species richness) for all treatments were comparable, falling within the range of 3122.40 to 3173.87, and there was no significant difference among the treatment groups. Nevertheless, the Shannon index values of bacterial communities varied significantly across the treatment groups. The TM treatment exhibited the highest value, followed by the TS, TR, and CT treatments. The fungal Chao1 index was significantly elevated in the TR treatment, whereas no significant differences were observed between the TS and TM treatments. The Shannon index exhibited the highest values in the TR treatment, while the CT treatment demonstrated the lowest values. These results indicate that the application of ryegrass enhanced the richness of the fungal community.

**Figure 3:**
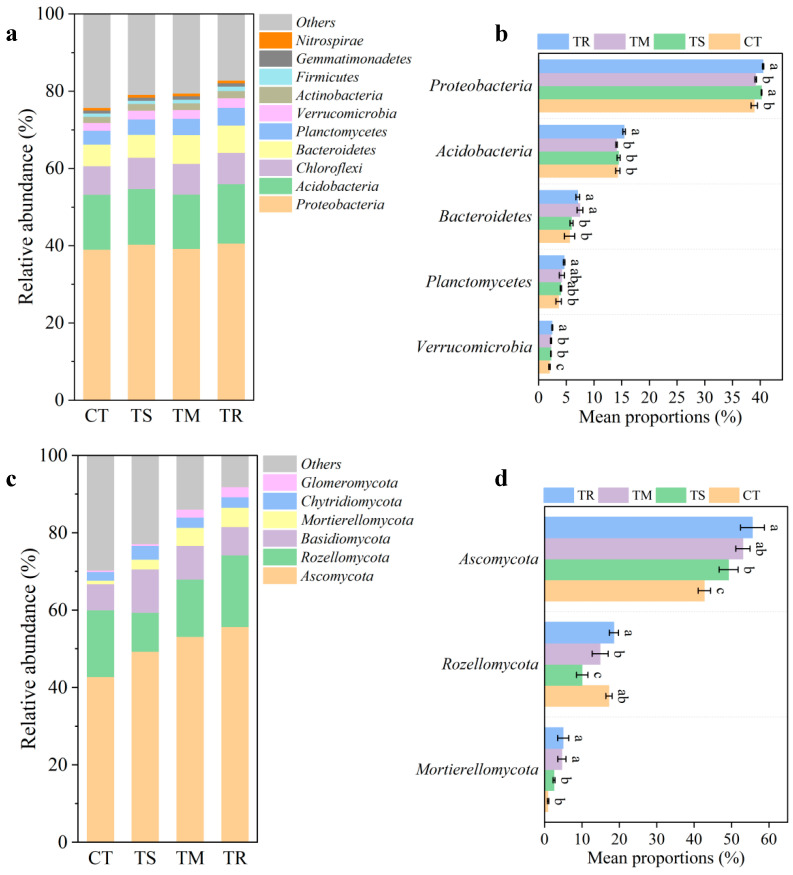
Relative abundance of soil bacteria (top ten, (**a**)) and fungi (top six, (**c**)) at the phylum level, as well as differential bacteria at the phylum level (**b**) and differential fungi at the phylum level (**d**); different lowercase letters denote significant differences among treatments.

Figure 3a depicts the relative abundance of bacterial phyla in soil samples under different treatments. At the phylum level, the composition of the bacterial community exhibited substantial variation across different treatments. Statistical analyses revealed specific and significant disparities among the treatments with respect to the dominant phyla. Proteobacteria was identified as the most abundant phylum. The relative abundance of Proteobacteria was significantly higher in the TM and TR treatments compared to the CT and TS treatments. The abundance of Acidobacteria was significantly greater in the TR treatment relative to the CT treatment. The relative abundances of Bacteroidetes, Planctomycetes, and Verrucomicrobia were all significantly lower in the CT group compared to the three green manure treatments.

The analysis of soil fungal communities revealed significant alterations in composition and abundance among the experimental treatments. The phylum *Ascomycota* predominated in all treatments; however, its relative abundance was significantly lower in the control group (CT) compared to the other treatments (TS, TM, and TR). The relative abundance of *Rozellomycota* also exhibited considerable variation. Specifically, treatments TS and TM demonstrated significantly reduced *Rozellomycota* abundance relative to the control, whereas treatment TR displayed the highest abundance. In contrast, *Mortierellomycota* proportions were consistently low across all groups, with treatments TR and TM exhibiting significantly higher proportions than CT and TS. Collectively, these findings indicate that the applied treatments exert a profound influence on soil fungal community structure, notably affecting the relative abundances of major fungal phyla such as *Ascomycota* and *Rozellomycota*.

**Figure 4:**
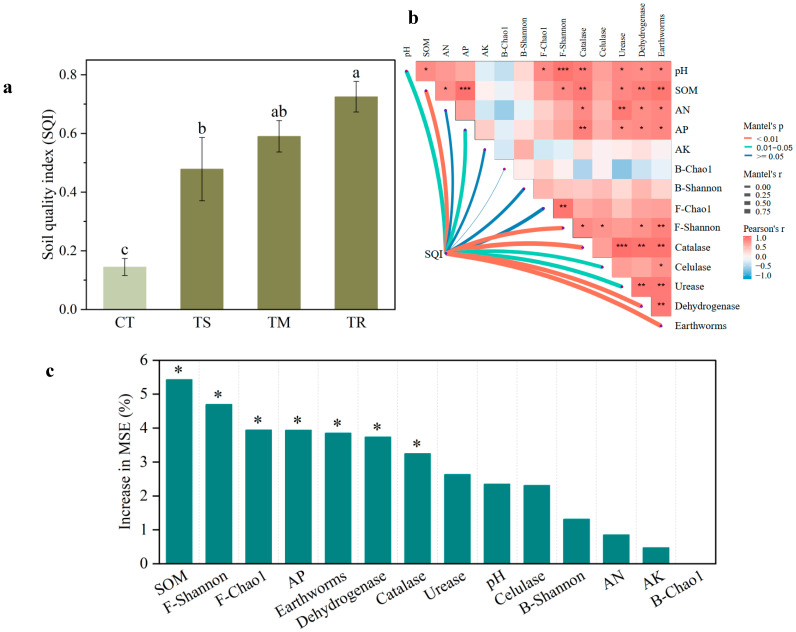
(**a**) Soil quality index (SQI) under different green manure management practices (CT, control with no green manure; TS, Spinach treatment; TM, Chinese milk vetch treatment; TR, Ryegrass treatment; different lowercase letters denote significant differences among treatments). (**b**) Mantel test results correlating SQI with soil factors. (**c**) Prediction of the importance of soil factors for the SQI. * *p* < 0.05, ** *p* < 0.01, *** *p* <0.001. B-Chao1, bacterial Chao1 index; B-Shannon, bacterial Shannon index; F-Chao1, fungal Chao1 index; F-Shannon, fungal Shannon index; SOM, soil organic matter; AN, available nitrogen; AP, available phosphorus; AK, available potassium.

As depicted in Figure 4a, all green manure treatments (TS, TM, and TR) significantly enhanced the Soil Quality Index (SQI) compared to the control group (CT). Compared with the CT group, the TS treatment resulted in a significant increase of 62.5% in SQI. The TM treatment exhibited a significantly higher improvement efficacy than the TS treatment, achieving a 38.5% increase relative to the TS treatment. In contrast, the TR treatment demonstrated the most substantial effect, with a 150% increment compared to the CT group, and also showed significant superiority over both the TS and TM treatments (increases of 53.8% and 11.1%, respectively). The differential increments in SQI across treatments in this study indicated significant variations in the efficiency of soil health improvement among different green manure species, with ryegrass exhibiting the strongest comprehensive improvement capacity. Thus, the sowing of ryegrass represented the most effective approach for achieving balanced and comprehensive enhancement of soil health.

As shown in Figure 4b, the results of the Mantel test indicated significant spatial associations between the Soil Quality Index (SQI) and soil factors. Soil organic matter (SOM), fungal diversity, catalase and dehydrogenase activities, and earthworm abundance exhibited highly significant and robust positive correlations with the Soil Quality Index (SQI) (*Mantel’s p* < 0.01). In contrast, soil pH, available phosphorus (AP), cellulase, and urease activities demonstrated significant positive correlations. Available nitrogen (AN) and available potassium (AK) exhibited comparatively modest yet statistically significant positive correlations. Moreover, a comparatively weak correlation was observed between the SQI and bacterial communities, with the B-Chao1 index even displaying a negative association. Collectively, soil biological indicators and key soil nutrient parameters exerted significant influence on the spatial distribution of soil quality.

As depicted in Figure 4c, the random forest predictive model was utilized to quantify the relative importance of different soil factors in predicting the Soil Quality Index (SQI). Soil organic matter (SOM) was identified as the most critical predictor of SQI, exhibiting the highest relative importance, while F-Shannon also demonstrated substantial importance for the SQI. Furthermore, soil biological indicators—including F-Chao1, available phosphorus (AP), earthworm abundance, and key enzymatic activities (dehydrogenase and catalase)—showed moderate predictive importance, underscoring that biological processes mediated by soil organisms significantly contribute to sustaining soil functionality. In contrast, variables such as soil urease, pH, cellulase, available nitrogen (AN), available potassium (AK), and bacterial richness provided relatively limited explanatory power within the predictive model. Collectively, the model outcomes highlight that soil fungal diversity, organic matter accumulation, and available phosphorus constitute the primary determinants of SQI.

## 4. Discussion

### 4.1. Green Manure Growth Enhances Soil Fertility and Enzyme Activity

Manifested through nutrient accumulation, treatments incorporating green manure significantly increased the contents of soil organic matter (SOM), available nitrogen (AN), and available phosphorus (AP). The marked rises in SOM, AN, and AP under the TR treatment indicated that green manure can effectively enhance soil organic matter content at its source, which serves as a core indicator for assessing soil fertility. A principal factor contributing to the 60.0% increase in available nitrogen observed in the TR treatment was the release of rhizodeposits from green manure root systems, which supplied abundant carbon sources for rhizosphere microorganisms. During the decomposition of these readily utilizable organic compounds, microorganisms accelerate the mineralization of soil organic matter, thereby converting organic nitrogen into inorganic forms accessible to plants and subsequently raising the soil available nitrogen content [25,26]. This process provides a substantial material foundation for crop growth. Concurrently, the accumulation of organic matter promotes the formation of soil aggregates, stimulates microbial mineralization of organic nitrogen in the soil, further strengthens the soil’s nitrogen supply capacity and productivity, improves the long-term availability of nitrogen, and reduces losses of organic matter through leaching or volatilization [27,28,29]. Long-term fertilization experiments have demonstrated that green manure can facilitate soil nitrogen cycling by enhancing biological nitrogen fixation [30]. In our study, all green manure treatments notably elevated the soil available phosphorus content. This outcome can be attributed to the transformation of phosphorus speciation from forms inaccessible to soil biota. Organic acids secreted by green manure root systems, such as citric acid and oxalic acid, are capable of chelating metal ions (e.g., Al^3+^, Fe^3+^) and dissolving insoluble phosphates in the soil, thereby mobilizing phosphorus, enhancing its bioavailability, and converting it into plant-available forms [4,31,32].

Furthermore, the differential responses of plant-available potassium to various treatments indicate species-specific nutrient cycling patterns. The results demonstrate that increases were observed in the TS and TM treatments. Microorganisms can utilize soil potassium to decompose residues with high carbon-to-nitrogen (C:N) ratios, thereby elevating their demand for potassium and other nutrients. This may lead to temporary potassium immobilization and a reduction in its availability to plants. Consequently, this underscores the necessity of optimizing green manure cultivation practices to prevent unintended nutrient limitations.

Regarding soil enzyme activity, the effects of green manure exhibited diversity (Figure 1). Our findings demonstrated that soil cellulase activity was not significantly affected by any green manure treatment. However, the results indicated that all green manure treatments significantly enhanced the activities of soil catalase, dehydrogenase, and urease. Among the evaluated treatments, the TR treatment exhibited the most marked influence on soil enzyme activities, indicating a functional linkage between green manure amendment and the promotion of nutrient cycling processes. Dehydrogenase activity represents a key indicator of total microbial metabolic activity [33]. The pronounced increase observed under this treatment implies that green manure effectively elevated the overall metabolic capacity of soil microorganisms. Furthermore, urease activity was significantly enhanced under the TR treatment, reflecting an accelerated rate of urea hydrolysis—a critical step within the nitrogen cycle. This stimulation of enzymatic activity can be attributed directly to the input of fresh organic matter, which supplies both substrates and a favorable micro-environment for soil microbial communities [34]. Carbon serves as both a carbon and energy source for microorganisms, thereby stimulating microbial proliferation and enzyme synthesis and ultimately enhancing soil biological activity [35,36]. Consequently, ryegrass emerges as the optimal choice for the rapid cultivation of soils with high biological activity and excellent nitrogen supply capacity. Furthermore, the Soil Quality Index (SQI) illustrated in Figure 4a functions as a comprehensive and precise indicator of soil health, enabling an integrated evaluation of soil quality and functionality [37]. Our findings confirm that alterations in green manure types modify soil biota, leading to differential integrated impacts on soil productivity and health status, which align with previous research [38]. The TR treatment exhibited a significant 150% increase in SQI compared to the CT group, indicating that the type of green manure can not only effectively enhance individual nutrient indicators, such as soil organic matter, but also synergistically improve various functions of the soil ecosystem, thereby positively influencing overall soil health.

Regarding the enhancement of soil biological activity, as evidenced by elevated enzyme activities (e.g., dehydrogenase, urease), our investigation interprets this phenomenon as indicative of improved soil ecological function. These enzymes play pivotal roles in nutrient cycling and organic matter decomposition, processes integral to soil health and fertility. Although heightened biological activity may occasionally arise as a stress response, within the context of green manure application, the observed increases coincide with marked improvements in soil physicochemical properties (soil organic matter, available nitrogen, available phosphorus) and earthworm abundance. This synergistic response suggests that the augmented activity reflects a more resilient and functionally robust soil ecosystem, rather than a stress-induced reaction. The incorporation of organic matter via green manure supplies readily available carbon substrates that stimulate microbial proliferation and enzyme synthesis, thereby stimulating biogeochemical processes essential for soil fertility.

### 4.2. Earthworm Populations Exhibit Positive Responses to Green Manure Amendment

Earthworm reproduction contributes to a positive feedback mechanism that enhances soil fertility. This study demonstrated that green manure application significantly increased both the abundance and biomass of earthworms in the amended soil (Figure 2), serving as robust biological indicators of improved soil health. These findings align with the typical response of earthworms to elevated soil organic matter, which provides a rich food source and a more favorable habitat environment.

Among the various experimental treatments, the TR treatment demonstrated significantly greater earthworm abundance and biomass relative to the control and other treatment groups. These results indicate that ryegrass can effectively promote the proliferation and growth of earthworm populations. Previous studies suggest that the well-developed root system of ryegrass likely provides enhanced food resources and habitat for earthworms, while its decomposition products may be more consistent with earthworm feeding preferences [39].

As illustrated in Figure 4b, the soil quality index (SQI) showed statistically significant positive correlations with both soil organic matter (SOM) content and earthworm abundance. This finding aligns with established literature confirming a positive relationship between soil organic matter levels and earthworm populations [9,11].

In contrast, applications of Chinese milk vetch (TM) and spinach (TS) also enhanced earthworm growth to varying extents; however, these effects were less significant compared to those observed under ryegrass treatment. The elevated earthworm biomass under TR can be partially attributed to the enhanced fungal richness (Table 2), given that fungi constitute a primary food source for earthworms. Previous research has demonstrated that the selection of green manure crop species can substantially affect the structure and functionality of the soil microbial community [40].

As pivotal soil ecosystem engineers, earthworms and soil microorganisms participate in intricate interactions that collectively enhance soil ecological functions. The distinct microbial community inhabiting the earthworm gut facilitates the further decomposition of organic matter and the generation of plant-available nutrients [41,42]. Moreover, microbial extracellular polymeric substances (EPS) constitute a significant component of soil organic carbon (SOC) and play a crucial role in soil structure formation and SOC stabilization. Earthworms can stimulate EPS production by promoting microbial activity and biomass, while also potentially accelerating EPS decomposition through their feeding activities [43]. This dualistic role highlights the complex yet substantial function of earthworms in regulating the soil organic carbon pool.

The feeding, digestion, and excretion processes of earthworms accelerate the decomposition of organic matter and nutrient cycling [44,45]. Processes mediated by the intestinal microbiota of earthworms and the production of nutrient-rich casts further enhance the decomposition rate of green manure and existing crop residues, such as rice straw [46]. Research indicates that earthworms can modulate soil microbial communities to improve straw degradation and increase the availability of nutrients for wheat [47]. This “biological tillage” incorporates organic matter into the soil matrix, thereby promoting nutrient cycling and soil structure development, which constitutes a critical element in sustainable soil management. Consequently, the selection of ryegrass as a green manure during the rice fallow period provides substantial benefits for optimizing the soil-improving functions of earthworms.

### 4.3. Green Manure Alters the Composition and Diversity of Soil Microbial Communities

Our present analysis demonstrated that green manure treatments induced specific modifications in the soil microbiome, with their overall impact on bacterial species richness remaining comparatively stable. These effects ranged from direct enhancements in microbial activity to alterations in community structure (Table 2, Figure 3). Notably, Chinese milk vetch treatments (TM) significantly elevated the bacterial Shannon index, indicating increased evenness and diversity within the bacterial community. The findings substantiate that, as a leguminous plant, Chinese milk vetch provides unique carbon and nitrogen sources to soil microorganisms through its inherent rhizobial activity, nitrogen fixation, and readily decomposable residues. This promotes the proliferation of a wider array of microbial taxa and substantially modifies the structure and function of the soil bacterial community, a result consistent with prior studies [30,48].

In the present study, the TR treatment significantly elevated fungal species richness (as quantified by the Chao1 index), while the TM treatment enhanced bacterial diversity. This phenomenon may be attributed to the more pronounced response of fungal richness to TR compared with TM or TS, which is likely attributable to the relatively lower pH sensitivity of fungi compared to bacteria. Consequently, the sustained input of substrates derived from ryegrass root exudates creates a more favorable environment for fungal proliferation [49]. These findings suggest that different types of green manure selectively enhance distinct microbial functional groups.

Furthermore, the results illustrated in Figure 4 demonstrated that ryegrass predominantly enhances the soil quality index (SQI) through the promotion of fungal diversity. The TR treatment concurrently and significantly elevated both soil organic matter (SOM) and fungal richness, thereby augmenting organic matter inputs and optimizing fungal community structure and microbial activity. This facilitation of nutrient transformation subsequently improved soil pH conditions and fostered earthworm growth and reproduction, establishing a positive feedback mechanism. This finding aligns with the concept of the “rhizosphere effect,” whereby diverse plant exudates generate distinct microbial niches [50].

For example, under the TM treatment, the elevated relative abundance of the Proteobacteria phylum—which comprises diverse functional groups such as nitrogen-fixing bacteria, nitrifiers, and denitrifiers—indicates that microbial communities adapt to stoichiometric imbalances. The soil C:N ratio serves as a key ecological stoichiometric parameter regulating microbial community structure, functional traits, and nutrient cycling processes. Furthermore, the addition of green manure modifies the inherent C:N ratio of the soil matrix [51]. When the soil C:N ratio deviates from the optimal stoichiometric requirements for microbial growth, microorganisms may modulate their community composition to preferentially select taxa capable of efficiently acquiring the limiting nutrient [52]. Elevated soil C:N ratios restrict microbial growth and enhance nitrogen immobilization, consequently promoting the dominance of microbial taxa that are adapted to nitrogen-limited conditions, such as fungi and actinomycetes [53,54]. Conversely, low soil C:N ratios restrict carbon utilization and induce nitrogen mineralization, thereby facilitating the rapid growth of r-strategist bacteria [53]. Furthermore, the C:N ratio regulates ecological processes such as soil organic matter decomposition, carbon sequestration, and greenhouse gas emissions by modulating microbial carbon use efficiency, enzyme activities, and the equilibrium between nitrogen mineralization and immobilization [55]. The effects of the C:N ratio on microbial communities can be further modulated by environmental factors, including nitrogen deposition and soil physicochemical properties [56].

Actinobacteria consistently demonstrated a high relative abundance across all green manure treatments. These bacterial taxa play a critical role in the decomposition of organic matter and the formation of humus. The shifts in fungal composition exhibited distinct variations, as evidenced by the relative reduction of Ascomycota in the CT group. However, the relative abundance of Ascomycota typically increased significantly following green manure application, potentially reflecting an alteration in the primary decomposition pathways, since different fungal phyla possess varying capacities for decomposing complex organic polymers [19].

The observation that green manure application enhances the abundance of beneficial microbial taxa, such as Actinobacteria and Proteobacteria, aligns with prior research [57]. The correlation between bacterial communities and soil properties, including pH, organic matter content, and specific available nutrients, underscores the interactive relationship between soil characteristics and microbial community dynamics. Furthermore, the availability of soil nutrients, particularly labile carbon and nitrogen, can constrain the abundance of Proteobacteria with increasing soil depth [58]. By providing differentiated organic substrates and altering the soil microenvironment, different green manures selectively enrich or suppress specific microbial groups, thereby influencing soil ecosystem functions [59,60]. These modifications in microbial community composition are functionally consequential, as they exert a direct influence on the rates and pathways of nutrient cycling and organic matter stabilization within the soil.

The observed enhancement in microbial diversity, specifically the augmentation of fungal richness under ryegrass treatment and bacterial diversity under Chinese milk vetch, is deemed advantageous within this agricultural framework. Although a nonspecific increase in diversity does not invariably yield positive outcomes, our findings demonstrate that these alterations correlate with an enrichment of beneficial microbial taxa (e.g., Proteobacteria, Actinobacteria), which are recognized for their contributions to nutrient cycling and soil structure formation. This implies the development of a more resilient and functionally diverse microbial community, capable of executing a broader spectrum of ecosystem services, including enhanced decomposition, nutrient mineralization, and disease suppression. The specific shifts observed are indicative of a healthier, more balanced microbial assemblage that underpins sustainable agricultural practices, as opposed to an uncontrolled proliferation that could prove detrimental.

## 5. Conclusions

In summary, the incorporation of green manure during the fallow period of paddy fields constitutes a highly effective practice for establishing a healthy and productive soil ecosystem. Our findings indicate that green manure cultivation significantly enhances soil organic matter and nutrient content, strengthens soil enzymatic activities, and increases the abundance of soil microorganisms and earthworms. These changes promote a positive feedback mechanism within the soil ecosystem and substantially improve the Soil Quality Index (SQI). Specifically, the Ryegrass treatment (TR) demonstrated consistently superior performance across multiple indicators, including a 150% increase in SQI. These improvements represent not merely quantitative enhancements but reflect fundamental alterations in soil ecological processes. For example, the elevated soil organic matter provides a sustained energy source for microbial communities, thereby driving enhanced nutrient mineralization and improved soil aggregation. The increased enzyme activities signify accelerated biogeochemical cycling, particularly for nitrogen and carbon, which are critical for plant growth and soil fertility. Moreover, the proliferation of earthworms and beneficial microbial taxa indicates a more robust and resilient soil food web, capable of efficiently decomposing organic residues and suppressing pathogenic organisms. These interconnected processes collectively contribute to a more stable and productive agroecosystem. From a practical standpoint, we strongly recommend the strategic selection and management of green manure species, particularly ryegrass, during paddy field fallow periods. This approach offers a sustainable and ecologically sound strategy for improving soil health, promoting biodiversity, and enhancing long-term agricultural productivity, thereby supporting more resilient and productive agroecosystems.

## Figures and Tables

**Table 1:** Properties of soil under different treatments.

Treatments	pH	SOM (g/kg)	AN (mg/kg)	AP (mg/kg)	AK (mg/kg)
CT	7.88 ± 0.25 b	35.89 ± 0.74 b	100.98 ± 5.71 b	88.67 ± 1.38 a	78.50 ± 3.54 a
TS	8.11 ± 0.01 ab	39.30 ± 2.82 ab	109.05 ± 17.14 b	112.11 ± 15.19 a	89.00 ± 1.41 a
TM	8.10 ± 0.01 ab	40.24 ± 2.49 ab	117.13 ± 5.71 b	120.90 ± 27.62 a	95.50 ± 4.95 a
TR	8.31 ± 0.08 a	44.13 ± 0.90 a	161.56 ± 0.00 a	134.57 ± 16.57 a	78.00 ± 15.56 a

Properties of soil under different treatments (CT, control with no green manure; TS, Spinach treatment; TM, Chinese milk vetch treatment; TR, Ryegrass treatment). SOM, soil organic matter; AN, available nitrogen; AP, available phosphorus; AK, available potassium. Values are means ± standard deviation. Values followed by different letters within a column indicate significant difference (*p* < 0.05) in Duncan’s test.

**Table 2:** Alpha diversity of microbial communities in soil under different treatments.

	Bacteria		Fungi	
Treatments	Chao1	Shannon	Chao1	Shannon
CT	3173.87 ± 43.01 a	6.74 ± 0.00 b	235.00 ± 28.28 b	3.25 ± 0.21 b
TS	3169.94 ± 153.39 a	6.81 ± 0.02 ab	263.22 ± 34.29 ab	3.61 ± 0.16 a
TM	3148.84 ± 9.79 a	6.85 ± 0.05 a	268.51 ± 15.17 ab	3.55 ± 0.03 a
TR	3122.40 ± 74.97 a	6.77 ± 0.01 b	279.92 ± 29.19 a	3.75 ± 0.18 a

Properties of soil under different treatments (CT, control with no green manure; TS, Spinach treatment; TM, Chinese milk vetch treatment; TR, Ryegrass treatment). Values are means ± standard deviation. Values followed by different letters within a column indicate significant difference (*p *< 0.05) in Duncan’s test.

## Data Availability

The original contributions presented in this study are included in the article. Further inquiries can be directed to the corresponding authors.

## Supplementary Materials

The following supporting information can be downloaded at: https://www.mdpi.com/article/10.3390/microorganisms14040870/s1, Figure S1: Soil morphology, soil section photo, and soil horizon sequence.

## Abbreviations

The following abbreviations are used in this manuscript:

CT	The control group
TS	Spinach treatment
TM	Chinese milk vetch treatment
TR	Ryegrass treatment
SOM	Soil Organic Matter
AN	Available Nitrogen
AP	Available Phosphorus
AK	Available Potassium
B-Chao1	Bacterial Chao1 index
B-Shannon	Bacterial Shannon index
F-Chao1	Fungal Chao1 index
F-Shannon	Fungal Shannon index
SQI	Soil Quality Index
PCA	Principal Component Analysis
