# Supplementary material for: Comparative Assessment of Diverse Green Manure Species for Enhancing Soil Quality, Microbial Communities, and Earthworm Growth in Fallow Paddy Fields
Source: Microorganisms. 2026 Apr 12;14(4):870. doi: 10.3390/microorganisms14040870 (PMC13119160; doi:10.3390/microorganisms14040870)
Supplement: Supplementary file 1 [file microorganisms-14-00870-s001.zip › microorganisms-4206115-supplementary.pdf]

Comparative Assessment of Diverse Green Manure Species for Enhancing Soil Quality, Microbial Communities, and Earthworm Growth in Fallow Paddy Fields

Lijuan Sun [1, 2, 3#], Zhenni Zhao [1, 2, 3#], Qin Qin [1, 2], Yafei Sun [1, 2], Shiyan Yang [1, 2], Xiaofeng Jiang [1, 2], Zhenglong Wang [4], Jun Wang [*], Yong Xue [1, 2, 3*]

[1] ECO-Environment Protection Research Institute, Shanghai Academy of Agricultural Sciences, Shanghai 201403, China.

[2] Shanghai Low Carbon Agricultural Engineering Technology Research Center, Shanghai 201403, China.

[3] School of Ecological Technology and Engineering, Shanghai Institute of Technology, Shanghai 201418, China.

[4] Shanghai Right Way Environmental Protection Technology Co., Ltd, Shanghai, 201208, China.

* Correspondence: (Jun Wang) junwang2018@163.com , (Yong Xue) exueyong@163.com

# Lijuan Sun and Zhenni Zhao contributed equally to this work.

Experimental Soil and its taxonomy and morphological characteristics

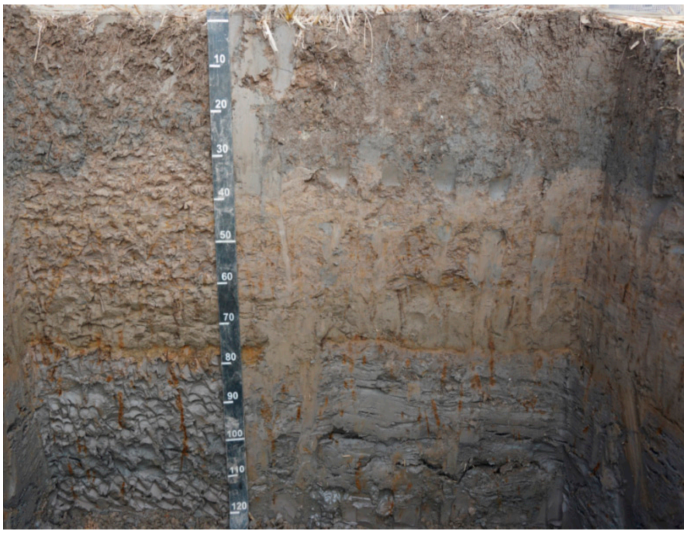

Soil Taxonomy

Soil Order: Paddy Soil

Suborder: Leached Paddy Soil

Great Group: Pale Muddy Paddy Soil

Soil Series: Sandy Clay Loam Pale Muddy Paddy Soil

Soil Horizon

Ap1 Horizon: 0–22 cm (Very Dark Grayish Brown)

Ap2 Horizon: 22–31 cm (Very Dark Brown)

Br1 Horizon: 31–63 cm (Dark Grayish Brown)

Br2 Horizon: 63–80 cm (Brown)

BCr Horizon: 80–120 cm (Very Dark Gray)

Figure S1. Soil morphology, soil section photo, and soil horizon sequence.

Morphological Characteristics

Ap1 horizon: 0–22 cm, no clay films, no mineral concretions or nodules, no cemented pan, moderately calcareous, no ferrolysis reaction, no alkalinization reaction, smooth clear boundary to underlying horizon; very dark grayish brown (10YR 3/2), silty clay loam, strongly developed fine angular blocky structure, loose, few iron oxide mottles, a moderate amount of medium roots, no clay films, no mineral concretions or nodules, no cemented pan, strongly calcareous, no ferrolysis reaction, no alkalinization reaction, smooth clear boundary to the underlying horizon.

Ap2 horizon: 22–31 cm, very dark brown (10YR 2/2), silty clay loam, strongly developed fine angular blocky structure, firm, few iron oxide mottles, few fine roots present, no clay films, no mineral concretions or nodules, no cemented pan, strongly calcareous, slight ferrolysis reaction, no alkalinization reaction, smooth clear boundary to the underlying horizon.

Br1 horizon: 31–63 cm, dark grayish brown (10YR 4/2), sandy loam, strongly developed fine angular blocky structure, firm, common iron–manganese oxide mottles, few fine roots, no clay films, no mineral concretions or nodules, no cemented pan, very strongly calcareous, no ferrolysis reaction, no alkalinization reaction, smooth clear boundary to the underlying horizon.

Br2 horizon: 63–80 cm, brown (10YR 5/3), sandy loam, strongly developed fine angular blocky structure, firm, common fine iron-manganese oxide mottles, no roots, no clay films, no mineral concretions or nodules, no cemented pan, very strongly calcareous, no ferrolysis reaction, no alkalinization reaction, smooth clear boundary to the underlying horizon.

BCr horizon: 80–120 cm, very dark gray (10YR 3/1), loam, strongly developed fine granular structure, firm, common fine iron-manganese oxide mottles, no roots.
